# Noonan syndrome and Turner syndrome patients respond similarly to 4 years’ growth-hormone therapy: longitudinal analysis of growth-hormone-naïve patients enrolled in the NordiNet® International Outcome Study and the ANSWER Program

**DOI:** 10.1186/s13633-015-0015-1

**Published:** 2015-09-08

**Authors:** Peter A. Lee, Judith L. Ross, Birgitte Tønnes Pedersen, Primoz Kotnik, John A. Germak, Henrik T. Christesen

**Affiliations:** Penn State College of Medicine, The Milton S. Hershey Medical Center, 500 University Dr., MC-H085, Hershey, PA 17033-0850 USA; Thomas Jefferson University, Philadelphia, PA USA; DuPont Hospital for Children, Wilmington, DE USA; Epidemiology, Novo Nordisk A/S, Søborg, Denmark; Department of Pediatric Endocrinology, University Children’s Hospital, University Medical Centre Ljubljana, Ljubljana, Slovenia; Novo Nordisk Inc, Princeton, NJ USA; Hans Christian Andersen Children’s Hospital, Odense University Hospital, Odense, Denmark

**Keywords:** Noonan syndrome, Turner syndrome, Human growth hormone

## Abstract

**Background:**

Turner syndrome (TS) and Noonan syndrome (NS) are distinct syndromes associated with short stature and other similar phenotypic features. We compared the responses to growth hormone (GH) therapy of TS and NS patients enrolled in the NordiNet® International Outcome Study (IOS) or the American Norditropin Studies: Web-Enabled Research (ANSWER) Program, which collect information on GH therapy in clinical practice.

**Methods:**

Repeated-measures regression analysis was performed on change in height standard deviation score (HSDS) and target-height-corrected HSDS, based on national normal references and treatment-naïve disease-specific references. Models were adjusted for baseline age and HSDS, and average GH dose. The study population was paediatric patients with TS and NS in the NordiNet® IOS and ANSWER Program. Longitudinal growth responses over 4 years were evaluated.

**Results:**

In 30 NS patients (24 males; baseline age 8.39 ± 3.45 years) and 294 TS patients (7.81 ± 3.22 years), 4-year adjusted ΔHSDS were +1.14 ± 0.13 and +1.03 ± 0.04, respectively (national references). Based on untreated, disease-specific references, 4-year adjusted ΔHSDS for NS and TS were +1.48 ± 0.10 and +1.79 ± 0.04. The analyses showed a significant increase in HSDS over time for both NS and TS (*P* < 0.0001). ΔHSDS in NS was higher with younger baseline age; ΔHSDS in TS was higher for patients with younger baseline age and higher GH dose.

**Conclusions:**

NS and TS patients responded well and similarly over 4 years of GH treatment.

**Electronic supplementary material:**

The online version of this article (doi:10.1186/s13633-015-0015-1) contains supplementary material, which is available to authorized users.

## Background

The genetic disorders Turner syndrome (TS) and Noonan syndrome (NS) are distinct clinical conditions sharing phenotypic similarities, including short stature [1–5].

TS affects at least one in 2500 live-born females [4]. Short stature is a prevalent feature, linked with haplo-insufficiency of the short-stature homeobox-containing (SHOX) gene [4]. TS girls usually achieve an adult height ~20–21 cm shorter than otherwise healthy women [3, 4]. The typical growth pattern is of growth retardation, with slow growth initially during infancy, subnormal growth rates in childhood, and absence of a pubertal growth spurt. TS girls usually have a normal pattern of GH secretion [4].

NS has a similar prevalence to TS (one in 1000–2500 live-born male and female births) [5]. Genetic mutations are identifiable in 70–80 % of NS patients [6], with missense mutations on the protein tyrosine phosphatase non-receptor type 11 gene (*PTPN11*) being the most frequently identified in around 50 % [5]. Some 50–70 % of NS patients have short stature [5]. The causes of poor growth in NS are complex. GH-secretory dynamics and insulin-like growth factor (IGF)-I levels in NS range from deficient to normal, and may reflect the genotypic heterogeneity of NS. Some NS patients have GH deficiency (GHD). Usually, individuals with NS are born with appropriate size for gestational age, but untreated children only reach median adult heights of 162.5 cm for men and 153.0 cm for women (European cohorts), i.e., adult heights averaging approximately –2 standard deviation scores (SDS) from the reference population [5, 7, 8].

Treatment with recombinant human GH is recommended for conditions associated with growth failure, including TS and NS [1–5, 9]; however, considerable variability of growth response to GH treatment has been reported across diagnostic categories [10–15]. GH treatment is not universally approved in short NS patients; more long-term data on the growth response in these patients are therefore merited.

Few studies have compared growth response to GH treatment over time between the two syndromes, and one published report comparing GH responses in TS and NS patients (US National Cooperative Growth Study [NCGS]) included NS patients with relatively high mean age (11.6 years) at treatment start [16].

The NordiNet® International Outcome Study (IOS) is a European-based registry (launched in 2006); the American Norditropin Studies: Web-Enabled Research (ANSWER) Program is a US-based registry (initiated in 2002). Both are long-term, observational studies designed to collect information on effectiveness and safety of the recombinant human GH product Norditropin® ([somatropin (rDNA origin) injection] Novo Nordisk, Bagsværd, Denmark) in real-world practice [17].

We report on 4-year longitudinal growth outcomes in paediatric NS and TS patients enrolled in the NordiNet® IOS and ANSWER Program.

## Methods

### Study design

The study designs of the ongoing NordiNet® IOS (ClinicalTrials.gov identifier: NCT00960128) and ANSWER Program (ClinicalTrials.gov identifier: NCT00615953) are described in detail elsewhere [17]. These non-interventional, real-world data studies include patients treated with Norditropin® as prescribed by their physicians. The study population considered in this paper comprises paediatric patients with a clinical diagnosis of NS or TS, who were GH-treatment naïve at enrolment and prescribed GH therapy with Norditropin®. The study databases use web-based platforms (NordiNet® and NovoNet®) for data capture in electronic Case Report Forms, providing automatic validation at data entry. The studies were conducted in accordance with the Declaration of Helsinki; patients provided written, informed consent and data were anonymised.

### Data extraction and statistical analyses

In the NordiNet® IOS and ANSWER Program, all clinical diagnoses were made by the treating physicians, according to standard practice, who also entered all patient information. Study protocol did not require genetic information. This report included all patients with an NS or TS diagnosis who had complete 4-year longitudinal data.

Key demographic and clinical characteristics, captured at baseline and during clinic visits, included: birth date; sex and clinical diagnosis; patient’s height; parents’ height; bone age; age at GH treatment initiation; GH dose and serum IGF-I levels [17]. Where available, pubertal status data were gathered – prepubertal status was defined based on clinical pubertal development (Tanner breast stage B1; testicular volume < 4 mL). Safety data were gathered, where available.

IGF-I values were measured locally and converted into IGF-I SDS based on age- and sex-related normative reference values [18]. Baseline data were extracted within a 3-month window before GH treatment started, and follow-up data at first-, second-, third- and fourth-year (±3 months) visits.

NS and TS children’s growth responses, from start through 4 years of GH treatment, were compared with national reference growth charts and the untreated disease-specific references for NS [7] and TS children [19–21]. The latter are hereafter referred to as the ‘Ranke’ [7, 19], ‘Cabrol’ [20] and ‘Westerlaken’ [21] reference populations.

Attainment of genetic height potential, i.e., target-height (TH)-corrected height standard deviation score (HSDS), defined as HSDS minus target HSDS, during 4 years of GH treatment was calculated (referred to hereafter as ‘TH-corrected’ data). Target height was determined by the corrected mid-parental height method (adding/subtracting 6.5 cm for boys/girls, respectively).

Changes in HSDS (ΔHSDS) from baseline were calculated, and growth response in NS and TS children were analysed and compared by a mixed linear model including repeated-measures. This was performed for three different responses: ΔHSDS based on national references; ΔHSDS based on disease-specific references; and ΔHSDS based on TH-corrected HSDS. To adjust for confounding factors, the data-model included covariates of age at treatment start, HSDS at baseline and average GH dose. For growth data based on disease-specific population references for NS and TS, HSDS and ΔHSDS are labelled ‘disease specific’. Otherwise, growth data for HSDS and ΔHSDS are based on national references of healthy children. Estimates adjusted for confounders are denoted ‘adjusted’.

Figures show estimated values (mean ± SE) obtained by mixed linear models including repeated measures and adjusted for confounders. Unadjusted estimates (mean ± SD) for HSDS and ΔHSDS are included in the Additional file 1: Table S1-S3.

## Results

### Patients

By November 2013, 30 NS patients (24 males, 6 females; baseline age 8.39 ± 3.45 years) and 294 female TS patients (baseline age 7.81 ± 3.22 years) had complete 4-year longitudinal data. At baseline, NS and TS patients did not differ significantly in age, HSDS and TH-corrected HSDS, bone age or IGF-I SDS (Table 1, unadjusted data). Observed baseline HSDS for NS was –2.64 ± 0.96 and for TS was –2.67 ± 0.88. Using untreated disease-specific reference populations (Ranke), baseline HSDS was –0.46 ± 0.89 in NS vs +0.30 ± 0.99 in TS (*P* < 0.001); baseline HSDS in TS was –0.20 ± 1.01 using the Westerlaken and –0.05 ± 1.03 using the Cabrol reference standard.

**Table 1:** Baseline demographic characteristics of Noonan syndrome (NS) and Turner syndrome (TS) patients with complete 4-year longitudinal data (unadjusted data)

Baseline characteristic	NS, mean ± SD		TS, mean ± SD	
	All patients	GHD subset^a^	All patients	GHD subset^a^
N, Sex (male:female)	*N* = 30 (24:6)	*n* = 8 (7:1)	*N* = 294 (0:294)	*n* = 21
Age, years (range)	8.39 ± 3.45 (2.38–14.29)	8.97 ± 4.19 (2.38–14.04)	7.81 ± 3.22 (0.51–15.23)	8.22 ± 3.21 (3.08–14.45)
HSDS	−2.64 ± 0.96	−2.61 ± 0.81	−2.67 ± 0.88	−2.84 ± 0.53
HSDS (Ranke)^b^ [7, 19]	−0.46 ± 0.89	−0.43 ± 0.85	0.30 ± 0.99	−0.07 ± 0.67
HSDS (Westerlaken) [21]	NA	NA	−0.20 ± 1.01	−0.53 ± 0.71
HSDS (Cabrol) [20]	NA	NA	−0.05 ± 1.03	−0.39 ± 0.70
Target HSDS	−0.43 ± 0.83	−0.81 ± 0.75 (*n* = 6)	−0.24 ± 1.02	−0.56 ± 0.89
TH-corrected HSDS^c^	−2.19 ± 1.14	−1.70 ± 1.19 (*n* = 6)	−2.46 ± 1.16	−2.27 ± 1.10
Bone age, years	6.92 ± 3.58	6.96 ± 4.92 (*n* = 6)	6.72 ± 3.03	7.56 ± 3.12 (*n* = 12)
Bone age – chronological age	−1.61 ± 1.22	−2.08 ± 1.17 (*n* = 6)	−1.31 ± 1.12	−1.49 ± 1.07 (*n* = 12)
Father’s height (cm)	176.19 ± 7.38	173.78 ± 6.71 (*n* = 6)	176.94 ± 7.89	172.85 ± 8.05
Mother’s height (cm)	161.53 ± 8.49	158.08 ± 4.96 (*n* = 6)	163.57 ± 6.96	162.18 ± 6.93
IGF-I SDS	−1.42 ± 1.45 (*n* = 19)	−2.52 ± 1.27 (*n* = 5)	−0.89 ± 1.51 (*n* = 147)	−1.21 ± 1.28 (*n* = 11)

*GHD* Growth hormone deficiency, *HSDS* Height standard deviation score, *NA* Not applicable, *TH* Target height, *SDS* Standard deviation score

^a^GHD defined by GH peak < 10 μg/L

^b^HSDS (Ranke) NS vs TS mean difference –0.76 (95% confidence interval –1.13, –0.39); *P* < 0.001

^c^TH-corrected HSDS refers to attainment of genetic height potential, i.e., parental-height-corrected HSDS – defined as HSDS minus target HSDS, during 4 years of GH treatment

At baseline, 17 NS patients (mean age 7.61 years) were prepubertal and two were pubertal/postpubertal (mean age 14 years). Information on pubertal status was missing for 11 NS patients. After 4 years’ GH treatment, 9 NS patients remained prepubertal (baseline mean age 5.37 years, chronological age 9.24 years); 12 were pubertal/postpubertal (mean age at baseline 11.13 years, chronological age 14.78 years). Information was missing for 9 NS patients. At baseline, 241 TS patients were prepubertal (mean age 7.73 years); 11 were pubertal/postpubertal (mean age 11.58 years). Information was missing for 42. After 4 years’ GH treatment, 143 TS patients were prepubertal (baseline mean age 5.84 years, chronological age 10.08 years); 106 were pubertal/postpubertal (baseline mean age 10.31 years, chronological age 14.29 years). Information was missing for 45.

A limited number of GH stimulation tests were performed at baseline – in 16 of 30 NS and 30 of 294 TS patients. Twenty-nine patients in the cohort met the criteria for GHD (peak GH < 10 μg/L: 8 of 16 NS (mean 4.70 μg/L) and 21 of 30 TS patients (mean 6.30 μg/L) (Table 1 for baseline characteristics).

Mean cumulative GH dose over 4 years was similar for NS and TS patients (48.85 ± 11.18 μg/kg/day and 47.64 ± 10.52 μg/kg/day, respectively). An increase in mean GH dose was observed over time in NS patients compared with baseline (*P* = 0.0478); no significant change occurred in TS patients (Table 2).

**Table 2:** Mean daily GH dose in Noonan syndrome (NS) and Turner syndrome (TS) patients over 4 years of GH treatment

	NS	TS
	GH dose μg/kg/day	GH dose μg/kg/day
	Mean ± SD (n)	Mean ± SD (n)
Baseline	44.32 ± 9.63 (27)	48.24 ± 13.20 (282)
Year 1	49.33 ± 11.82 (30)	48.20 ± 12.03 (286)
Year 2	50.86 ± 12.19 (27)	47.90 ± 12.41 (287)
Year 3	50.46 ± 13.04 (28)	47.47 ± 13.57 (288)
Year 4	49.78 ± 13.56 (27)	46.50 ± 12.90 (267)

### Growth responses

**Fig. 1:**
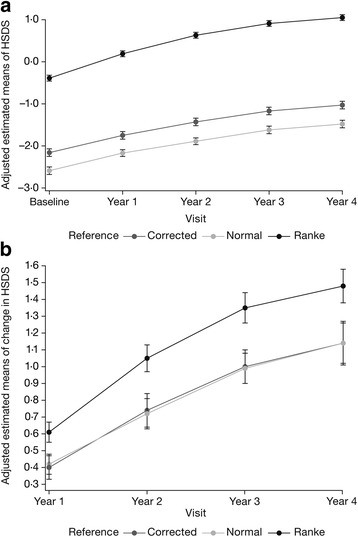
**a** Comparison of adjusted estimated means of Noonan syndrome (NS) height standard deviation score (HSDS) references (± SE): target height (TH)-corrected HSDS values, normal-height standard deviation score values, and HSDS values based on the Ranke reference [19]. **b** Comparison of adjusted estimated means of change in NS HSDS references (±SE): TH-corrected and normal ΔHSDS, and ΔHSDS values based on Ranke reference [19]

In NS patients, HSDS values based on normal national references were lower than TH-corrected HSDS values (Fig. 1a) (Additional file 1: Table S1 for unadjusted estimated means for HSDS). Baseline HSDS (mean ± SE) was –2.59 ± 0.09, increasing to –1.48 ± 0.09 at year 4. Using the Ranke NS reference, baseline HSDS was –0.39 ± 0.07, increasing to 1.05 ± 0.07 after 4 years’ GH treatment.

TH-corrected ΔHSDS and ΔHSDS were almost identical, while disease-specific ΔHSDS values based on the Ranke NS reference population were higher than ΔHSDS and TH-corrected ΔHSDS (Fig. 1b) (Additional file 1: Table S2 for unadjusted ΔHSDS estimates). The ΔHSDS (mean ± SE) was 0.42 ± 0.06 at year 1, increasing to 1.14 ± 0.13 at year 4. The disease-specific Ranke ΔHSDS (mean ± SE) was 0.61 ± 0.06 at year 1, increasing to 1.48 ± 0.10 at year 4.

**Fig. 2:**
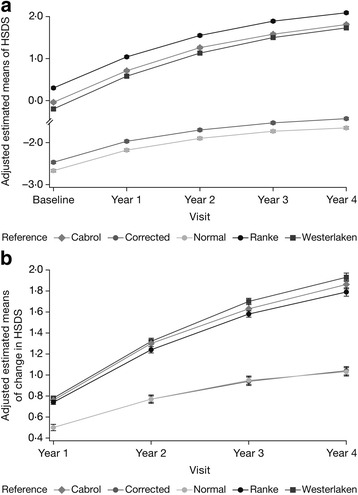
**a** Comparison of adjusted estimated means of Turner syndrome (TS) height standard deviation score (HSDS) references (± SE): target height (TH)-corrected HSDS values, normal-height standard deviation score values, and HSDS values based on the reference populations of Ranke [19], Cabrol [20] and Westerlaken [21]. **b** Comparison of adjusted estimated means of change in TS HSDS references (± SE): TH-corrected and normal ΔHSDS, and ΔHSDS values based on the reference populations of Ranke [19], Cabrol [20] and Westerlaken [21]

Figure 2 depicts HSDS data for the TS patients. HSDS and TH-corrected HSDS values were similar (Fig. 2a) (Additional file 1: Table S1 for unadjusted estimated means). Baseline HSDS (mean ± SE) was –2.67 ± 0.03, increasing to –1.65 ± 0.03 at year 4. Baseline HSDS (mean ± SE) based on the Ranke TS reference was 0.30 ± 0.03, increasing to 2.09 ± 0.03 after 4 years’ GH treatment. Similar trends were observed using the Cabrol TS and Westerlaken TS references.

The ΔHSDS values based on disease-specific TS reference values were greater than the ΔHSDS and TH-corrected ΔHSDS based on normal population references (Fig. 2b, Additional file 1: Table S2). The ΔHSDS (mean ± SE) was 0.50 ± 0.03 at year 1, increasing to 1.03 ± 0.04 at year 4. The disease-specific Ranke ΔHSDS (mean ± SE) was 0.74 ± 0.02 at year 1, and 1.79 ± 0.04 at year 4. Using the Westerlaken reference, mean ± SE ΔHSDS was 0.78 ± 0.02 at year 1, and 1.93 ± 0.04 at year 4. Mean ± SE ΔHSDS (Cabrol) for TS was similar: 0.76 ± 0.02 at year 1, and 1.86 ± 0.04 at year 4.

**Fig. 3:**
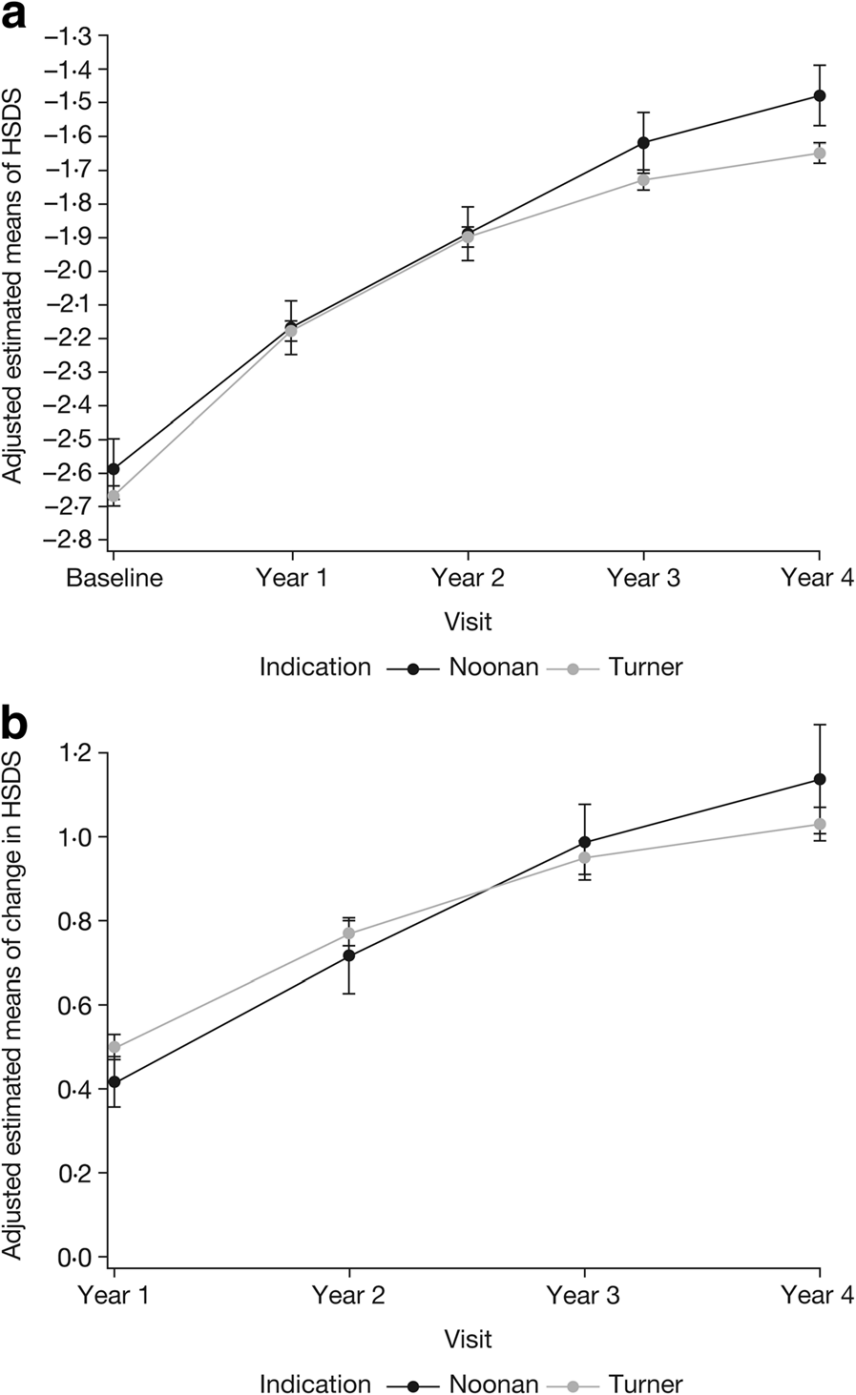
**a** Adjusted estimated means of height standard deviation score (HSDS; ± SE) by indication and visit year; adjusted for age at treatment start, HSDS at baseline, and average GH dose. **b** Adjusted estimated means of the change in HSDS, ΔHSDS (± SE) from baseline by indication and visit year; adjusted for age at treatment start, HSDS at baseline and average GH dose

NS and TS patients responded similarly to GH therapy over time (Fig. 3a and b). Figure 3a provides the HSDS estimated means ± SE per indication based on the normal national references adjusted for age at treatment start, HSDS at baseline and average GH dose. The adjustment only changed the values marginally from the unadjusted, crude data (Additional file 1: Table S1). Figure 3b depicts the estimated means (± SE) for ΔHSDS from baseline to 4-year follow-up for each indication. This figure illustrates the similar response in the NS and TS patients; no significant difference between the indications was found (*P* = 0.6281). The adjustment changed the values only marginally (Additional file 1: Table S2).

Disease-specific ΔHSDS (Ranke) from baseline showed a significant increase from baseline per visit-year for each indication (NS: years 1–2 and years 2–3 [*P* < 0.0001], and years 3–4 [*P* = 0.0271]; TS: years 1–2, years 2–3, and years 3–4 [all *P* < 0.0001]). Data analysis and modelling using the reference populations Westerlaken and Cabrol showed similar trends to the findings based on the Ranke reference population assessments (data not shown).

Additional analysis of ΔHSDS adjusting for baseline age, HSDS at baseline and average relative GH dose confirmed no difference between linear growth responses to GH therapy at any visit according to indication (TS or NS) (*P* > 0.05). Further analysis determined that baseline age and years on treatment had a significant impact on the change in HSDS based on the normal national reference in NS patients (*P* = 0.0037 and *P* < 0.0001, respectively), while all the modelled parameters (baseline HSDS, age at baseline, years on treatment and average GH dose) had a significant effect on responses in TS patients (*P* < 0.0001, *P* < 0.0001, *P* < 0.0001 and *P* = 0.0265, respectively; parameter estimates not shown).

Percentages of patients in the NS and TS cohorts with HSDS within and below the normal range at baseline and each year of follow-up are shown in Table 3. At baseline, 80 % of NS and 82 % of TS patients were below –2 HSDS; by year 4, this decreased to 23 and 35 %, respectively.

**Table 3:** Patients with height below/within normal ranges by indication

	Noonan syndrome patients		Turner syndrome patients	
	HSDS < –2	HSDS > –2	HSDS < –2	HSDS > –2
	*n* (%)	*n* (%)	*n* (%)	*n* (%)
Baseline	24 (80)	6 (20)	240 (82)	54 (18)
Year 1	16 (53)	14 (47)	166 (56)	128 (44)
Year 2	15 (50)	15 (50)	127 (43)	167 (57)
Year 3	9 (30)	21 (70)	113 (38)	181 (62)
Year 4	7 (23)	23 (77)	102 (35)	192 (65)

*HSDS* Height standard deviation score

Analysis of change in IGF-I SDS, adjusted for baseline IGF-I SDS, age at treatment start and average GH dose, showed no significant differences in change in IGF-I SDS over time between the two patient populations (Additional file 1: Table S3).

### Safety data

The following adverse events were reported for the study population included in this paper. One patient with TS, enrolled in 2006, showed progression in her scoliosis in 2010. In another TS patient, four concurrent events were reported (headache, adenoidectomy, paracentesis, tonsillectomy), with only headache considered possibly related to GH treatment. Other events reported in individual TS patients included epiphysiolysis observed after 6.5 years on treatment (considered probably treatment related, with a dose reduction made in therapy), a case of osteomyelitis after 9 years on treatment and a case of developmental glaucoma after 2.5 years on treatment (all considered possibly treatment related). A case of cardiac failure in a TS patient after 7 years on treatment was considered unlikely related to GH treatment and a case of genital haemorrhage, after 4 years’ treatment in another TS patient, was of unknown cause. One NS patient reported two episodes of headache after 1 year of treatment, which were both considered treatment related.

## Discussion

The NordiNet® IOS and ANSWER Program data on long-term outcomes for patients with NS and TS demonstrate that the 2 patient groups responded well and similarly during 4 years’ GH treatment – measured by mean ΔHSDS and TH-corrected ΔHSDS based on normal national references.

Choice of reference population for growth in TS and NS patients is important to the interpretation of the magnitude of the growth response, with a mean 4-year adjusted ΔHSDS varying from +1.14 (normal reference) to +1.48 (Ranke) in NS patients, and from +1.03 (normal reference) to +1.93 (Westerlaken) in TS patients. Effectiveness of treatment was similar, whichever disease-specific reference population was applied to TS, attesting to the robustness of our results.

Mean heights in untreated NS follow the third percentile during the first years of life, generally declining further at the normal age of puberty onset [7, 19, 22]. In 45 adult NS patients, spontaneous height gain from age 8 years reached +0.57 SDS in boys and +1.00 SDS in girls, using the Prader reference, and was interpreted as the result of delayed puberty in NS [23].

Short-term GH therapy increases growth velocity, while longer therapy results in more modest gains in adult height [1, 2, 5]. Much of the data on GH treatment in NS come from observational studies, in which GH therapy has been commonly initiated at an older age (often aged 10 years when HSDS may be as low as –3.0), using lower GH doses than those typically recommended for TS [8, 15, 16, 24, 25]. Preliminary data from the ANSWER Program reported that, in NS subjects with mean baseline age of 9.2 years, GH treatment for 4 years resulted in mean ΔHSDS of +1.33 (from –2.65 to –1.32), with no gender differences, for patients receiving a mean dose of 47 μg/kg/day at baseline and 59 μg/kg/day at year 4 [8].

The NordiNet® IOS and ANSWER Program data for 4 years of longitudinal therapy presented here demonstrate a mean ΔHSDS of +1.14 at 4 years’ treatment in NS patients. This analysis compares favourably with a study in which a mean ΔHSDS of +0.8 (from –2.7 to –1.9 SDS) was reported after 3 years of GH treatment [26]. In comparison, mean baseline HSDS in a group of eight control NS patients not treated with GH was –2.7, with a mean HSDS of –2.4 at year 3 [26]. In our NS patients, the mean adjusted ΔHSDS was +1.00 at year 3 and +1.14 at year 4.

Although not directly comparable, due to a shorter treatment period and younger age at treatment start, our 4-year growth response data in NS patients with a mean age of 8.39 years at enrolment appears to be in line with those reported in the NCGS, which included 65 patients who had a mean age of 11.6 years at enrollment, and were treated with a mean GH dose of 0.33 mg/kg/week (0.047 mg/kg/day). In patients achieving near adult height, the NCGS reported an increase of +1.4 in HSDS (from –3.5 to –2.1) after a mean duration of 5.6 years of GH treatment, whereas in the total NS patient group, an increase of +1.2 in HSDS (from –3.2 to –2.0) was achieved after a mean duration of 5.3 years of GH treatment [16].

In our study, we found more robust growth responses in NS patients than those reported from the Kabi International Growth Study (KIGS) database, in which a 3-year longitudinal prepubertal cohort of 73 NS patients (median age 7.7 years at start of therapy) had a total increase in HSDS of +0.8 after 3 years (increment of +0.54, +0.13 and +0.13 in years 1, 2 and 3, respectively), compared with the ∆HSDS of +1.00 at year 3 in the current study [13].

Although it is unclear whether responses to GH therapy may be influenced by the genetic causes of NS, it has been suggested that the 50 % of NS children with *PTPN11* gene mutations show reduced responses to GH therapy compared with those with other mutations [8, 24, 27–31]. Due to the observational nature of our studies, in which genetic data were not prospectively collected for NS patients, it was not possible to analyse our dataset according to patient genotype.

We observed that, based on TH SDS, parental heights for NS patients may differ more from the normal population than is the case for TS parental heights. As adults with NS are often fertile, this may suggest that some parents of NS patients may have undiagnosed NS.

The NordiNet® IOS and ANSWER Program 4-year data also highlight the benefits of long-term GH therapy in girls with TS, and add to the body of evidence on the effects of GH therapy on growth and final height achievement in TS patients, including recent reports suggesting good growth responses to GH in TS girls, even when treatment is not initiated until the age of 12 years [32–35].

Previous reports from the ANSWER Program suggest that gains in height in TS patients during short-term GH treatment are highly predictive of longer-term results. In one report, the continuation of GH treatment for ≥ 3 years resulted in 62.3 % of the TS patients achieving an HSDS within the normal population range [32]. Our study included 294 TS patients with a relatively young mean age of 7.81 years at baseline, with 65 % of patients achieving normal HSDS at year 4. This was not a final adult height in our cohort, so more may achieve normal height SDS with ongoing treatment.

Despite almost identical proportions of NS and TS patients with HSDS above –2 at baseline (20 and 18 %, respectively), 77 % of NS, but only 65 % of TS patients, were above –2 for HSDS after 4 years. This may be attributed to the observed increase in mean GH dose in NS patients from baseline to year 4. In addition, the covariate modelling analysis showed a significant effect of average GH dose on growth response in TS patients. These findings further support safe treatment optimization, including individualized GH dose titration consistent with currently approved product labels.

We observed that NS patients responded well to long-term GH therapy and showed linear growth comparable to that in GH-treated TS patients. This may provide evidence to challenge the belief that NS patients may be less responsive to GH therapy, or show initial responses that wane over time. The dose and young age at initiation of GH therapy may have contributed to the good growth responses observed for NS patients in our study.

Our study included baseline measures of GH and assessed IGF-I levels during GH treatment in a small proportion of patients; the results suggest no differences in change in IGF-I over time in both populations.

Strengths of this analysis include the real-world and longitudinal nature of the study, collecting data from many TS and NS patients in several countries and allowing for longer follow-up than may be possible in a clinical-trial setting. Other strengths include use of disease-specific references and the application of a model adjusting for confounding factors. This cohort includes NS patients who started treatment at a younger age than is typically reported in the literature.

The observational nature of the NordiNet® IOS and ANSWER Program data limits analysis of the outcomes, particularly regarding assessment of outcomes according to genetic diagnoses of NS and TS, and analysis of data in relation to the pubertal status of patients during the study. Likewise, the limited number of GH stimulation tests means it is not possible to determine the differential proportion of NS and TS patients with GHD and the impact of GHD on results. The data allow for a comparison of outcomes for TS and NS patients but the smaller sample size for NS may hamper the ability to see year-on-year differences as clearly as were noted in the TS cohort.

## Conclusions

In conclusion, the NordiNet® IOS and ANSWER Program 4-year data demonstrate that, in real-world clinical practice, NS and TS patients can achieve good long-term responses to GH therapy, and responded similarly to 4 years of GH therapy. The 4-year data show a significant increase in HSDS over time for both NS and TS patients, measured by mean ΔHSDS and TH-corrected ΔHSDS based on normal national reference values and mean ΔHSDS based on disease-specific references. In both populations, the ΔHSDS was higher for patients with younger baseline age. The data add to understanding of the long-term responses to GH therapy. Notably, the 4-year period reported from the international cohort of NS and TS patients in this study is longer than typical patient follow-up in clinical studies, thus complementing and supplementing current knowledge. In addition, patients in clinical studies are often older at treatment outset than described in our dataset. Thus our report demonstrates growth benefits from early treatment initiation and continuing GH treatment in both NS and TS patients as might occur in everyday clinical practice.

## Additional file

Additional file 1: Table S1. Estimated means (unadjusted) of HSDS for Noonan syndrome and Turner syndrome patients per year. **Table S2.** Estimated means (unadjusted) of ΔHSDS for Noonan syndrome and Turner syndrome patients from baseline. **Table S3.** Change in IGF-I SDS over time. (DOC 174 kb)

## Abbreviations

ANSWER: American Norditropin Studies: Web-Enabled Research
GH: Growth hormone
GHD: Growth hormone deficiency
HSDS: Height standard deviation score
IGF: Insulin-like growth factor
IOS: International Outcome Study
KIGS: Kabi International Growth Study
NA: Not applicable
NCGS: National Cooperative Growth Study
NS: Noonan syndrome
SDS: Standard deviation scores
SHOX: Short-stature homeobox-containing
TH: Target height
TS: Turner syndrome

## References

[1] Chacko E, Graber E, Regelmann MO, Wallach E, Costin G, Rapaport R (2012). Update on Turner and Noonan syndromes. Endocrinol Metab Clin North Am..

[2] Chacko EM, Rapaport R (2012). Short stature and its treatment in Turner and Noonan syndromes. Curr Opin Endocrinol Diabetes Obes..

[3] Baxter L, Bryant J, Cave CB, Milne R. Recombinant growth hormone for children and adolescents with Turner syndrome. Cochrane Database Syst Rev. 2007:CD003887. 10.1002/14651858.CD003887.pub2 17253498

[4] Bondy CA (2007). Care of girls and women with Turner syndrome: a guideline of the Turner Syndrome Study Group. J Clin Endocrinol Metab..

[5] Romano AA, Allanson JE, Dahlgren J, Gelb BD, Hall B, Pierpont ME (2010). Noonan syndrome: clinical features, diagnosis, and management guidelines. Pediatrics..

[6] Noonan JA, Kappelgaard AM (2015). The efficacy and safety of growth hormone therapy in children with noonan syndrome: a review of the evidence. Horm Res Paediatr..

[7] Ranke MB, Heidemann P, Knupfer C, Enders H, Schmaltz AA, Bierich JR (1988). Noonan syndrome: growth and clinical manifestations in 144 cases. Eur J Pediatr..

[8] Lee PA, Ross J, Germak JA, Gut R (2012). Effect of 4 years of growth hormone therapy in children with Noonan syndrome in the American Norditropin Studies: Web-Enabled Research (ANSWER) Program® registry. Int J Pediatr Endocrinol..

[9] Richmond E, Rogol AD (2010). Current indications for growth hormone therapy for children and adolescents. Endocr Dev..

[10] Blum WF, Ross JL, Zimmermann AG, Quigley CA, Child CJ, Kalifa G (2013). GH treatment to final height produces similar height gains in patients with SHOX deficiency and Turner syndrome: results of a multicenter trial. J Clin Endocrinol Metab..

[11] Lee PA, Germak J, Gut R, Khutoryansky N, Ross J (2011). Identification of factors associated with good response to growth hormone therapy in children with short stature: results from the ANSWER Program®. Int J Pediatr Endocrinol..

[12] Pasquino AM, Passeri F, Municchi G, Segni M, Pucarelli I, Larizza D (1996). Final height in Turner syndrome patients treated with growth hormone. Horm Res..

[13] Raaijmakers R, Noordam C, Karagiannis G, Gregory JW, Hertel NT, Sipila I (2008). Response to growth hormone treatment and final height in Noonan syndrome in a large cohort of patients in the KIGS database. J Pediatr Endocrinol Metab..

[14] Rosenfeld RG, Frane J, Attie KM, Brasel JA, Burstein S, Cara JF (1992). Six-year results of a randomized, prospective trial of human growth hormone and oxandrolone in Turner syndrome. J Pediatr..

[15] Ranke MB (2009). Noonan syndrome: growth to growth hormone - the experience of observational studies. Horm Res..

[16] Romano AA, Dana K, Bakker B, Davis DA, Hunold JJ, Jacobs J (2009). Growth response, near-adult height, and patterns of growth and puberty in patients with noonan syndrome treated with growth hormone. J Clin Endocrinol Metab..

[17] Höybye C, Sävendahl L, Christesen HT, Lee P, Pedersen BT, Schlumpf M (2013). The NordiNet® International Outcome Study and NovoNet® ANSWER Program®: rationale, design, and methodology of two international pharmacoepidemiological registry-based studies monitoring long-term clinical and safety outcomes of growth hormone therapy (Norditropin®). Clin Epidemiol..

[18] Brabant G, von zur Mühlen A, Wüster C, Ranke MB, Kratzsch J, Kiess W (2003). Serum insulin-like growth factor I reference values for an automated chemiluminescence immunoassay system: results from a multicenter study. Horm Res.

[19] Ranke MB, Stubbe P, Majewski F, Bierich JR (1988). Spontaneous growth in Turner's syndrome. Acta Paediatr Scand Suppl..

[20] Cabrol S, Saab C, Gourmelen M, Raux-Demay MC, Le Bouc Y (1996). Turner syndrome: spontaneous growth of stature, weight increase and accelerated bone maturation. Arch Pediatr.

[21] Rongen-Westerlaken C, Corel L, van den Broeck J, Massa G, Karlberg J, Albertsson-Wikland K (1997). Reference values for height, height velocity and weight in Turner's syndrome. Swedish Study Group for GH treatment. Acta Paediatrica.

[22] Otten BJ, Noordam C (2009). Growth in Noonan syndrome. Horm Res..

[23] Binder G, Grathwol S, Von LK, Blumenstock G, Kaulitz R, Freiberg C, et al. Health and quality of life in adults with Noonan syndrome. J Pediatr. 2012;161:501–5. 10.1016/j.jpeds.2012.02.043 22494877

[24] Dahlgren J (2009). GH therapy in Noonan syndrome: review of final height data. Horm Res..

[25] Osio D, Dahlgren J, Wikland KA, Westphal O (2005). Improved final height with long-term growth hormone treatment in Noonan syndrome. Acta Paediatrica..

[26] MacFarlane CE, Brown DC, Johnston LB, Patton MA, Dunger DB, Savage MO (2001). Growth hormone therapy and growth in children with Noonan's syndrome: results of 3 years' follow-up. J Clin Endocrinol Metab..

[27] Choi JH, Lee BH, Jung CW, Kim YM, Jin HY, Kim JM (2012). Response to growth hormone therapy in children with Noonan syndrome: correlation with or without PTPN11 gene mutation. Horm Res Paediatr..

[28] De Rocca Serra-Nédélec A, Edouard T, Treguer K, Tajan M, Araki T, Dance M (2012). Noonan syndrome-causing SHP2 mutants inhibit insulin-like growth factor 1 release via growth hormone-induced ERK hyperactivation, which contributes to short stature. Proc Natl Acad Sci U S A..

[29] Noordam C, Peer PG, Francois I, De Schepper J, van den Burgt I, Otten BJ (2008). Long-term GH treatment improves adult height in children with Noonan syndrome with and without mutations in protein tyrosine phosphatase, non-receptor-type 11. Eur J Endocrinol..

[30] Ferreira LV, Souza SA, Arnhold IJ, Mendonca BB, Jorge AA (2005). PTPN11 (protein tyrosine phosphatase, nonreceptor type 11) mutations and response to growth hormone therapy in children with Noonan syndrome. J Clin Endocrinol Metab..

[31] Bertelloni S, Baroncelli GI, Dati E, Ghione S, Baldinotti F, Toschi B (2013). IGF-I generation test in prepubertal children with Noonan syndrome due to mutations in the PTPN11 gene. Hormones (Athens).

[32] Ross J, Lee PA, Gut R, Germak J (2011). Impact of age and duration of growth hormone therapy in children with Turner syndrome. Horm Res Paediatr..

[33] Ross JL, Quigley CA, Cao D, Feuillan P, Kowal K, Chipman JJ (2011). Growth hormone plus childhood low-dose estrogen in Turner's syndrome. N Engl J Med..

[34] Ranke MB, Lindberg A, Brosz M, Kaspers S, Loftus J, Wollmann H (2012). Accurate long-term prediction of height during the first four years of growth hormone treatment in prepubertal children with growth hormone deficiency or Turner Syndrome. Horm Res Paediatr..

[35] Bettendorf M, Inta IM, Doerr HG, Hauffa BP, Mehls O, Ranke MB (2013). Height gain in Ullrich-Turner syndrome after early and late growth hormone treatment start: results from a large retrospective German study and potential basis for an individualized treatment approach. Horm Res Paediatr..

