# Supplementary material for: Noonan syndrome and Turner syndrome patients respond similarly to 4 years’ growth-hormone therapy: longitudinal analysis of growth-hormone-naïve patients enrolled in the NordiNet® International Outcome Study and the ANSWER Program
Source: Int J Pediatr Endocrinol. 2015 Sep 8;2015(1):17. doi: 10.1186/s13633-015-0015-1 (PMC4562101; doi:10.1186/s13633-015-0015-1)
Supplement: Additional file 1: Table S1. — Estimated means (unadjusted) of HSDS for Noonan syndrome and Turner syndrome patients per year. Table S2. Estimated means (unadjusted) of ΔHSDS for Noonan syndrome and Turner syndrome patients from baseline. Table S3. Change in IGF-I SDS over time. (DOC 174 kb) [file 13633_2015_15_MOESM1_ESM.doc]

Supplemental Tables to Lee et al:

**Noonan syndrome and Turner syndrome patients respond similarly to 4 years’ growth-hormone therapy: longitudinal analysis of growth-hormone-naïve patients enrolled in the NordiNet International Outcome Study and the ANSWER Program**

*In the main manuscript, we report data as attainment of genetic height potential, i.e., target-height (TH)-corrected height standard deviation score (HSDS), defined as HSDS minus target HSDS, during 4 years of GH treatment (referred to as ‘TH-corrected’ data). Target height was determined by the corrected mid-parental height method (adding/subtracting 6.5 cm for boys/girls, respectively). In the main manuscript, models were adjusted for baseline age and HSDS, and average GH dose. Here, we present unadjusted data as supplemental tables.*

**Table S1 Estimated means (unadjusted) of HSDS for Noonan syndrome and Turner syndrome patients per year**

|  | **Noonan syndrome Turner syndrome** | | |
| --- | --- | --- | --- |
|  | **Unadjusted HSDS (SD)** | **Unadjusted HSDS (SD)** | |
| Normal | | | |
| Baseline | –2.64 (0.96) | | –2.67 (0.88) |
| Year 1 | –2.20 (0.84) | | –2.17 (0.89) |
| Year 2 | –1.91 (0.99) | | –1.90 (0.97) |
| Year 3 | –1.64 (0.96) | | –1.73 (1.00) |
| Year 4 | –1.46 (1.01) | | –1.65 (1.00) |
| TH-corrected | | | |
| Baseline | –2.19 (1.14) | | –2.46 (1.16) |
| Year 1 | –1.78 (1.10) | | –1.96 (1.18) |
| Year 2 | –1.44 (1.15) | | –1.70 (1.22) |
| Year 3 | –1.18 (1.13) | | –1.53 (1.25) |
| Year 4 | –1.01 (1.06) | | –1.43 (1.21) |
| Ranke [18] | | | |
| Baseline | –0.46 (0.89) | | 0.30 (0.99) |
| Year 1 | 0.15 (0.84) | | 1.03 (1.01) |
| Year 2 | 0.60 (0.97) | | 1.53 (1.05) |
| Year 3 | 0.88 (1.03) | | 1.87 (1.09) |
| Year 4 | 1.03 (1.08) | | 2.06 (1.13) |
| Cabrol [19] | | | |
| Baseline | – | | –0.05 (1.03) |
| Year 1 | – | | 0.71 (1.03) |
| Year 2 | – | | 1.25 (1.10) |
| Year 3 | – | | 1.58 (1.11) |
| Year 4 | – | | 1.80 (1.13) |
| Westerlaken [20] | | | |
| Baseline | – | | –0.20 (1.01) |
| Year 1 | – | | 0.58 (1.02) |
| Year 2 | – | | 1.12 (1.06) |
| Year 3 | – | | 1.49 (1.10) |
| Year 4 | – | | 1.72 (1.13) |

HSDS: Height standard deviation score; SD: Standard deviation.

**Table S2 Estimated means (unadjusted) of ΔHSDS for Noonan syndrome and Turner syndrome patients from baseline**

|  | **Noonan syndrome Turner syndrome** | | |
| --- | --- | --- | --- |
|  | **Unadjusted ΔHSDS (SD)** | **Unadjusted ΔHSDS (SD)** | |
| Normal | | | |
| Year 1 | 0.43 (0.44) | | 0.50 (0.49) |
| Year 2 | 0.73 (0.54) | | 0.77 (0.61) |
| Year 3 | 1.00 (0.53) | | 0.94 (0.71) |
| Year 4 | 1.18 (0.65) | | 1.02 (0.78) |
| TH-corrected | | | |
| Year 1 | 0.41 (0.42) | | 0.50 (0.49) |
| Year 2 | 0.75 (0.53) | | 0.77 (0.61) |
| Year 3 | 1.01 (0.55) | | 0.94 (0.71) |
| Year 4 | 1.18 (0.65) | | 1.03 (0.77) |
| Ranke [18] | | | |
| Year 1 | 0.61 (0.32) | | 0.74 (0.34) |
| Year 2 | 1.06 (0.45) | | 1.24 (0.49) |
| Year 3 | 1.34 (0.50) | | 1.58 (0.60) |
| Year 4 | 1.49 (0.51) | | 1.78 (0.71) |
| Cabrol [19] | | | |
| Year 1 | – | | 0.76 (0.40) |
| Year 2 | – | | 1.30 (0.57) |
| Year 3 | – | | 1.63 (0.70) |
| Year 4 | – | | 1.85 (0.78) |
| Westerlaken [20] | | | |
| Year 1 | – | | 0.78 (0.35) |
| Year 2 | – | | 1.32 (0.50) |
| Year 3 | – | | 1.69 (0.60) |
| Year 4 | – | | 1.92 (0.70) |

ΔHSDS: Change in height standard deviation score; SD: Standard deviation.

**Table S3 Change in IGF-I SDS over time**

| **Change in IGF-I SDS from baseline** | **Noonan syndrome** | | **Turner syndrome** | |
| --- | --- | --- | --- | --- |
|  | **N** | **Mean (SD)** | **N** | **Mean (SD)** |
| 1 year | 6 | 1.67 (1.42) | 81 | 2.12 (1.62) |
| 2 years | 12 | 1.63 (1.39) | 85 | 1.72 (1.73) |
| 3 years | 11 | 1.46 (2.18) | 90 | 1.89 (1.78) |
| 4 years | 11 | 1.75 (1.86) | 89 | 2.04 (2.06) |

IGF: Insulin-like growth factor; SD: Standard deviation; SDS: Standard deviation score.
